# Obstetrical use of intravenous immunoglobulin: A single-centre retrospective study

**DOI:** 10.1016/j.htct.2025.106225

**Published:** 2025-12-06

**Authors:** Roy Khalife, Bonnie Niu, Iris Perelman, Darine El-Chaâr, Dean Fergusson, Alan Karovitch, Johnathan Mack, Melanie Tokessy, Kathryn E. Webert, Alan Tinmouth

**Affiliations:** a Department of Medicine, University of Ottawa, Ottawa, ON, Canada; b Ottawa Hospital Research Institute, Ottawa, ON, Canada; c Faculty of Medicine, University of Ottawa, Ottawa, ON, Canada; d Department of Obstetrics and Gynecology, University of Ottawa, Ottawa, Ontario, Canada; e Eastern Ontario Regional Laboratory Association, Ottawa Hospital, Ottawa, Ontario, Canada; f Department of Medicine and the Department of Molecular Medicine and Pathology, McMaster University, Hamilton, Ontario, Canada; g Canadian Blood Services, Ontario, Canada; h Department of Medicine, McGill University, Montreal, QC, Canada

**Keywords:** Immunoglobulin, Pregnancy, Thrombocytopenia, ITP, Transfusion medicine

## Abstract

**Introduction:**

Intravenous immunoglobulin is widely used for various conditions but faces challenges such as limited supply, high cost, and substantial off-label use. Obstetrical intravenous immunoglobulin use remains underexplored, despite its relevance to maternal and neonatal care and resource management.

**Methods:**

This single-center retrospective cohort study examined intravenous immunoglobulin administration in 136 pregnancies (122 patients) from 2007–2020, focusing on adherence to Health Canada licensed indications and Ontario Immunoglobulin Utilization Management Guidelines.

**Results:**

Maternal thrombocytopenia (56.6 %) and treatment for fetal/neonatal alloimmune thrombocytopenia (16.2 %) were the most common indications, accounting for 16.9 % and 64.3 % of total intravenous immunoglobulin volume, respectively. Intravenous immunoglobulin use represented 1.6 % of the center's total consumption during the study period, with notable non-adherence to guidelines in 38.2 % (Health Canada) and 17.6 % (provincial guidelines) of pregnancies.

**Conclusion:**

Findings highlight the need for optimized intravenous immunoglobulin use in obstetrics and future research to ensure safety, efficacy, and evidence-based guidance in clinical practice and policy.

## Introduction

Intravenous immunoglobulin (IVIG), derived from pooled human plasma, is used to treat immunodeficiencies, autoimmune diseases, and potentially other conditions [1]. However, IVIG is in limited supply, has a high cost, and considerable off-label use, which is often not supported by strong evidence of benefits [1,2]. These challenges are pronounced in obstetrics, where balancing maternal and fetal health complicates treatment decisions.

Although IVIG use in obstetrics is generally reserved for specific scenarios or refractory cases, it may be favored over other options anecdotally due to the lack of alternatives and perceived safety. The failure to include pregnant women in randomized controlled trials examining IVIG use in labelled indications [3, 4, 5, 6, 7, 8, 9], and the absence of high-quality studies for off-label obstetrical indications [10,11], leave significant gaps in evidence-based guidance. Existing literature largely overlooks IVIG applications in obstetrics [2,12, 13, 14, 15], limiting understanding of its scope and potential misuse. This understanding is essential not only for optimizing patient care but also for ensuring the judicious use of a scarce resource.

This study assesses the obstetrical use of IVIG against established guidelines to inform policy, practice, and future research, which can help enhance obstetrical care and ensure stewardship of an expensive and limited resource. The objectives are two-fold: [1] to assess the frequency, dose, and indications of IVIG use in pregnancy, and [2] to assess concordance of IVIG use with the approved Canadian indications and the approved conditions of the Ontario Immunoglobulin Utilization Management Guidelines.

## Methods

A retrospective cohort study was conducted using administrative data from The Ottawa Hospital Data Warehouse complemented by chart reviews from electronic health records to evaluate IVIG use in pregnancy. The study population comprised all pregnant women who received IVIG between 2007 and 2020 and delivered at the Ottawa Hospital. Data were collected on IVIG volumes, regimens, indications, and timing of administration during pregnancy. Health Canada licensed indications, which include primary immunodeficiencies, secondary immunodeficiencies, chronic lymphocytic leukemia, immune thrombocytopenia, chronic inflammatory demyelinating polyneuropathy, Guillain Barre Syndrome, and multifocal motor neuropathy were used to examine guideline adherence [16]. In addition, appropriateness was assessed using the Ontario Immunoglobulin Utilization Management Guidelines, which include both licensed and non-licensed indications for IVIG as approved for provincial use [17]. Non-licensed indications could include conditions such as fetal/neonatal alloimmune thrombocytopenia (F/NAIT), and hemolytic disease of the fetus and newborn (HDFN) [17]. Data were analyzed using descriptive statistics. The study was approved by Ottawa Health Science Network Research Ethics Board (CRRF 2826/Protocol 20210315-01H).

## Results

### Overall use and trends

**Figure 1:**
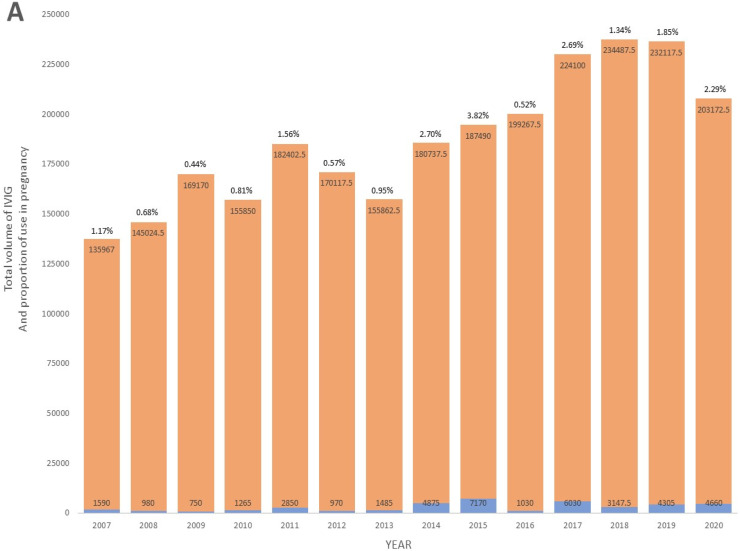

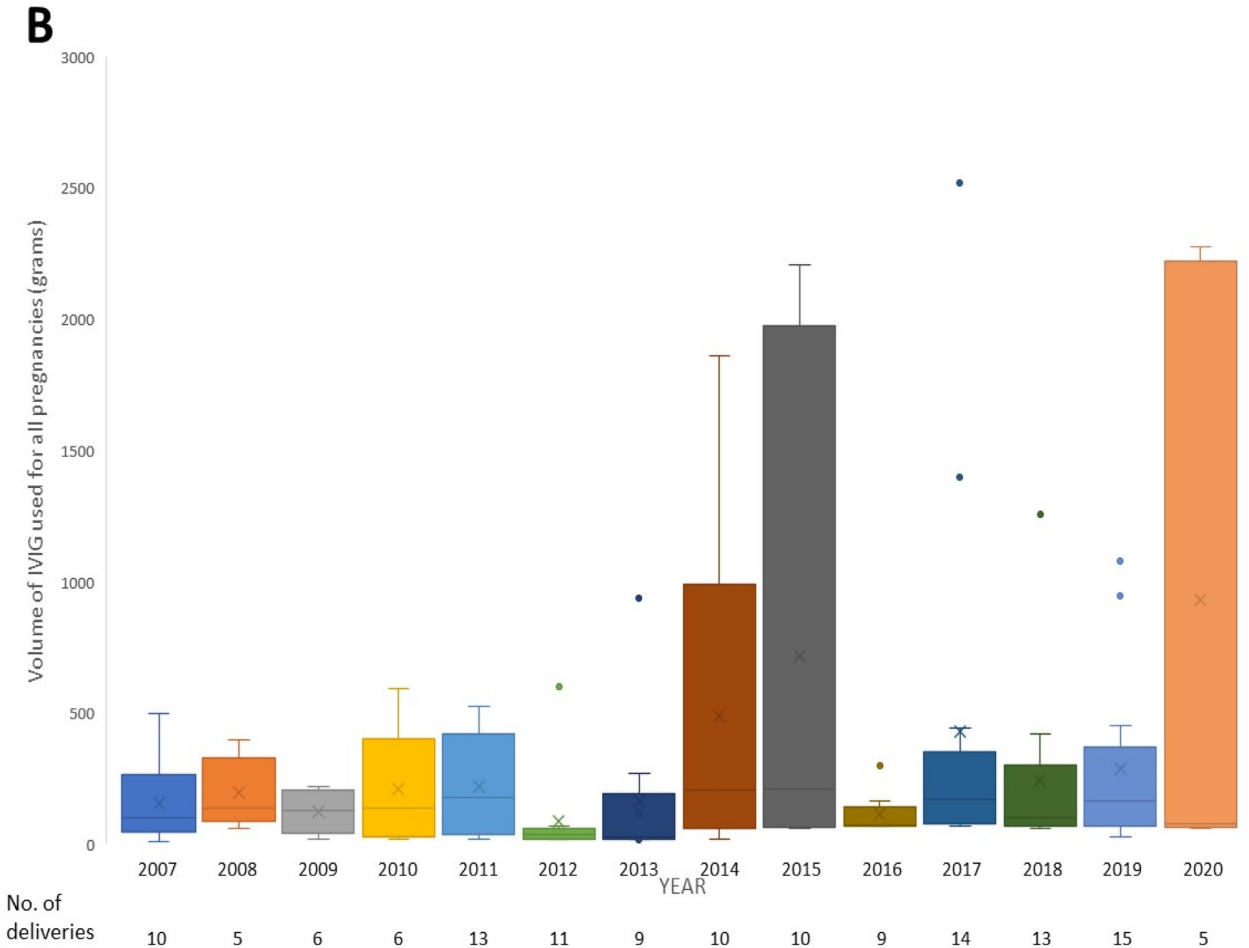
Temporal trends in IVIG administration from 2007-2020. (A) Total volume of IVIG administered in pregnancy and for the total population at our center, including proportion ( %) of IVIG volume used in pregnancy. (B) Box and whisker plots of the volume of IVIG use per pregnancy.

From 2007 to 2020, a total of 122 pregnant patients representing 136 deliveries were treated with IVIG during their pregnancy at the Ottawa Hospital. Cumulatively, these patients used 41,107.50 grams of IVIG. The volume accounted for 1.6 % of the total IVIG consumption at the center over this period. While the total IVIG usage at the Ottawa Hospital increased during the period of the study, the relative proportion of IVIG used in pregnancy also increased, with greater obstetrical use seen in the latter period of the study (Figure 1A). Overall, the annual mean proportion of IVIG volume used in pregnancy relative to the total population was 1.53 % (Standard deviation [SD]: 1.02). The annual mean volume of IVIG used in pregnancy was 2936.25 grams (SD: 2129.82), while the annual mean volume for the total population at the center was 183,983.32 grams (SD: 31,527.03 grams). Specific years exhibiting peaks in IVIG use in pregnancy were predominantly driven by a higher number of F/NAIT cases, where pregnant patients received weekly doses of IVIG for the entire second and third trimesters (Figure 1B).

### Indications for intravenous immunoglobulin use

The most prevalent indications for IVIG administration in pregnancy were related to hematologic conditions. Specifically, maternal thrombocytopenia was identified in 56.6 % (77/136) of deliveries, and antenatal therapy for F/NAIT was noted in 16.2 % (22/136) of deliveries. Other less common reasons, outlined in Table 1, included neurologic conditions (9.6 %), rheumatologic conditions (4.4 %), dermatologic conditions (2.9 %), obstetrical indications (2.2 %), immunodeficiencies (1.5 %), and renal conditions (0.7 %).

**Table 1:** Indication, volume, and dosing of IVIG administration for all deliveries.

Diagnosis	No. of deliveries n (%)	Total volume of IVIG administered grams (%)	Median dose of IVIG in grams (IQR 25^th^, 75^th^)	Frequency of IVIG administration
**Hematologic**	**107 (78.7)**	**35132.5 (85.5)**	**80 (60, 272.5)**	
Maternal isolated thrombocytopenia (Gestational thrombocytopenia, Immune thrombocytopenia	77 (56.6)	6952.5 (16.9)	70 (40, 90)	Single*
Antenatal therapy for Fetal/Neonatal Alloimmune Thrombocytopenia	22 (16.2)	26435 (64.3)	1015 (542.5, 1938.75)	Recurrent*
Antiphospholipid syndrome / Antiphospholipid antibodies	5 (3.7)	730 (1.8)	120 (100, 220)	Recurrent
Maternal red cell antibodies / Prevention of Hemolytic Disease of the Fetus and Newborn	3 (2.2)	1015 (2.5)	300 (280, 377.5)	Recurrent
**Neurologic**	**13 (9.6)**	**2230 (5.4)**	**165 (115, 180)**	
Myasthenia Gravis	5 (3.7)	695 (1.7)	175 (70, 175)	Recurrent*
Guilain-Barre Syndrome	3 (2.2)	350 (0.9)	115 (110 – 125)	Single
Chronic inflammatory demyelinating polyneuropathy	2 (1.5)	465 (1.1)	N/A	Single
Multiple Sclerosis	2 (1.5)	440 (1.1)	N/A	Recurrent
Small fiber polyneuropathy	1 (0.7)	280 (0.7)	N/A	Recurrent
**Rheumatologic**	**6 (4.4)**	**1365 (3.3)**	**220 (165, 290)**	
Maternal Anti-Ro Antibodies	4 (2.9)	1025 (2.5)	280 (200, 336.25)	Recurrent*
Rheumatoid Arthritis	1 (0.7)	180 (0.4)	N/A	Recurrent
Autoimmune necrotizing myositis	1 (0.7)	160 (0.4)	N/A	Recurrent
**Dermatologic**	**4 (2.9)**	**740 (1.8)**	**190 (165, 210)**	
Idiopathic Angioedema Urticaria	1 (0.7)	180 (0.4)	N/A	Recurrent
Pemphigoid Gestationis	2 (1.5)	440 (1.1)	N/A	Recurrent
Undiagnosed recurrent cutaneous eruptions	1 (0.7)	120 (0.3)	N/A	Recurrent
**Obstetrical**	**3 (2.2)**	**905 (2.2)**	**300 (280, 322.5)**	
Chronic Villitis	2 (1.5)	645 (1.6)	N/A	Recurrent
Repeated Implantation Failure	1 (0.7)	260 (0.6)	N/A	Recurrent
**Immunodeficiencies**	**2 (1.5)**	**315 (0.8)**	N/A	
Selective IgA deficiency	1 (0.7)	35 (0.1)	N/A	Single
Secondary Immunodeficiency (Hypogammaglobulinemia)	1 (0.7)	280 (0.7)	N/A	Recurrent
**Renal**	**1 (0.7)**	**420 (1.0)**		
Acute antibody-mediated rejection in renal transplant	1 (0.7)	420 (1.0)	N/A	Recurrent

⁎ Indicates the predominant frequency of IVIG administration for the listed indication.

### Intravenous immunoglobulin utilization

In terms of IVIG consumption, the antenatal treatment of F/NAIT accounted for the majority (64.3 %) of the total IVIG used in all pregnancies. This translated to 26,435 grams with a median of 1015 grams per pregnancy (Interquartile Range [IQR]: 542.5-1938.75 grams). Maternal thrombocytopenia followed, accounting for a total of 6,952.50 grams (16.9 %) used in all pregnancies and a median of 70 grams per pregnancy (IQR: 40-90 grams).

### Guideline adherence

Regarding the congruency of IVIG use with labelled Health Canada indications, 38.2 % (52/136) of the pregnancies received IVIG for off-label indications. This use for indications not approved by Health Canada, which includes F/NAIT, represented a substantial portion of the total volume of IVIG use in pregnancies, amounting for 33,025 grams (80.3 % of the total volume used). Other off-label indications under Health Canada included Myasthenia Gravis, Multiple Sclerosis, repeated Implantation Failure, Antiphospholipid syndrome, Rheumatoid arthritis, Pemphigoid Gestationis, Anti-Ro antibodies, HDFN, Antibody-mediated rejection (renal transplant), Chronic Villitis, Small fiber polyneuropathy, Idiopathic Angioedema, and Autoimmune Necrotizing Myositis.

In contrast, only 17.6 % (24/136) of pregnancies receiving IVIG, accounting for 5,475 grams (13 % of total volume used), did not adhere to the approved indications in the Ontario Immunoglobulin Utilization Management Guidelines. These conditions included Pemphigoid Gestationis, Idiopathic angioedema, Antiphospholipid antibodies, Anti-Ro antibodies, Autoimmune Myositis, Multiple Sclerosis, repeated implantation failure and Chronic Villitis.

## Discussion

This study offers a comprehensive picture of the patterns and scope of obstetrical use of IVIG, an area less explored in existing literature [2,13,14,18]. It demonstrates that IVIG use for obstetrical patients at the Ottawa Hospital has increased over the study period but accounts for only a small fraction of the overall IVIG consumption. The primary indications for IVIG administration during pregnancy included hematologic conditions, notably maternal thrombocytopenia, and the treatment of F/NAIT. A considerable portion of IVIG use did not align with the approved Health Canada indications, and a smaller but still important proportion did not align with the Ontario Immunoglobulin Utilization Management Guidelines. This difference is due to F/NAIT being an off-label Health Canada indication but appropriate use in Ontario guidelines. Overall, the off-label use suggests a potential for optimizing its application in obstetrical care.

Maternal thrombocytopenia and prevention of F/NAIT accounted for a substantial portion of IVIG use in this study cohort and may be important clinical scenarios necessitating further research. Thrombocytopenia occurs in about 10 % of pregnancies but rarely requires treatment [19]. In our experience, IVIG may be preferentially administered at the clinician’s discretion to avoid corticosteroid exposure with the aim of improving the platelet count over certain thresholds for labor and delivery particularly to allow for neuraxial anesthesia (generally a platelet count >70-80×10^9^/L), despite the lack of evidence to suggest meaningful clinical benefits for the mother or newborn [20,21]. As for the prevention of F/NAIT, IVIG appears to be effective based on small observational studies [11] and has achieved consensus as the treatment of choice despite the lack of high-quality evidence [22]. Such practices raise questions about the broader clinical decision-making processes guiding IVIG use for maternal thrombocytopenia and F/NAIT, particularly in the context of balancing efficacy, safety, and resource allocation.

The findings of the current study complement other studies in non-obstetrical settings that have documented high rates of reliance on IVIG for various conditions without high-quality evidence [1,14,15,18,23,24]. The proportion of non-adherence to guidelines in this study, accounting for 38.2 % of deliveries in terms of Canadian indications and 17.6 % in terms of Ontario guidelines, underscores a potential area for improvement in clinical practice. While the upward trend of IVIG use and the divergence from licensed indications and/or guidelines could reflect a growing recognition of the obstetrical and non-obstetrical indications and evolving understanding of the therapeutic roles of IVIG [11,21,25], it also raises concerns about resource utilization and the need for ongoing surveillance to ensure that IVIG is used appropriately and sustainably [1,15,26]. IVIG stewardship programs that involve an intermediary healthcare professional to monitor, review and guide IVIG administration have shown great promise for optimizing adherence to guidelines, and reducing inappropriate administration of IVIG and associated costs without negatively impacting patient care [23,24]. Stewardship programs that relied primarily on order request forms and handouts of clinical practice guidance had little to no influence on IVIG use [1].

The strengths of this study include its comprehensive data collection spanning over a decade and its focus on a large, diverse population served by a major Canadian tertiary care and academic institution. From this dataset, it was possible to conduct a detailed analysis of IVIG usage patterns and guideline adherence. However, the retrospective nature of the study limits the possibility to fully assess the clinical contexts leading to off-label IVIG use. Additionally, the single center focus may restrict the generalizability of the findings. This study also does not capture different practice patterns across centers, including center-specific approval processes for IVIG.

In conclusion, the present study sheds light on important aspects of IVIG use in pregnancy, highlighting areas of both adherence and deviation from licensed indications and established guidelines. These findings underscore the need for stewardship programs to optimize IVIG use in pregnancy, ensuring that this valuable resource is used effectively and responsibly in clinical practice. Several questions remain, particularly regarding the mechanisms driving off-label IVIG use in pregnancy and its clinical outcomes. Future research should aim to fill these gaps by exploring the safety, efficacy, and cost-effectiveness of IVIG in obstetrical care, especially for conditions lacking alternative treatments. Prospective studies and randomized controlled trials involving pregnant women are essential to establish evidence-based guidelines for IVIG use in this population, ensuring both maternal and fetal well-being while maintaining resource stewardship.

## Author contributions

RK, BN, IP, and AT performed the research. RK, DEC, DF, AK, JM, KW, and AT designed the research study and grant proposal. IP and MT contributed essential data. RK, BN and AT analyzed the data. RK wrote the paper. All authors revised the paper critically and approved the submitted and final versions.

## Funding statement

This study was funded by the Canadian Blood Services’ Blood Efficiency Accelerator Program.

## Ethics approval

The study was approved was approved by Ottawa Health Science Network Research Ethics Board (CRRF 2826/Protocol 20210315-01H).

## Data availability statement

The data that support the findings of this study are available from the corresponding author.

## Conflicts of interest

The authors do not have any conflict of interest to declare pertaining to this study.
